# Favipiravir (T-705) Protects IFNAR^−/−^ Mice against Lethal Zika Virus Infection in a Sex-Dependent Manner

**DOI:** 10.3390/microorganisms9061178

**Published:** 2021-05-29

**Authors:** Keesha Matz, Jackson Emanuel, Julie Callison, Don Gardner, Rebecca Rosenke, Reinaldo Mercado-Hernandez, Brandi N. Williamson, Heinz Feldmann, Andrea Marzi

**Affiliations:** 1 Laboratory of Virology, Division of Intramural Research, National Institute of Allergy and Infectious Diseases, National Institutes of Health, Hamilton, MT 59840, USA; matzkeesha@gmail.com (K.M.); jackson.emanuel@charite.de (J.E.); callisonj@niaid.nih.gov (J.C.); reinaldomercado1@gmail.com (R.M.-H.), brandi.williamson@nih.gov (B.N.W.), feldmannh@niaid.nih.gov (H.F.); 2 Rocky Mountain Veterinary Branch, Division of Intramural Research, National Institute of Allergy and Infectious Diseases, National Institutes of Health, Hamilton, MT 59840, USA; dgard0213@gmail.com (D.G.); rosenker@niaid.nih.gov (R.R.)

**Keywords:** flavivirus, ZIKV, mouse model, sex bias, ribavirin, antiviral, nucleoside analog

## Abstract

Zika virus (ZIKV), a member of the *Flaviviridae* family, is an important human pathogen that has caused epidemics in Africa, Southeast Asia, and the Americas. No licensed treatments for ZIKV disease are currently available. Favipiravir (T-705; 6-fluoro-3-hydroxy-2-pyrazinecarboxamide) and ribavirin (1-(β-D-Ribofuranosyl)-1*H*-1,2,4-triazole-3-carboxamide) are nucleoside analogs that have exhibited antiviral activity against a broad spectrum of RNA viruses, including some flaviviruses. In this study, we strengthened evidence for favipiravir and ribavirin inhibition of ZIKV replication in vitro. Testing in IFNAR^−/−^ mice revealed that daily treatments of favipiravir were sufficient to provide protection against lethal ZIKV challenge in a dose-dependent manner but did not completely abrogate disease. Ribavirin, on the other hand, had no beneficial effect against ZIKV infection in this model and under the conditions examined. Combined treatment of ribavirin and favipiravir did not show improved outcomes over ribavirin alone. Surprisingly, outcome of favipiravir treatment was sex-dependent, with 87% of female but only 25% of male mice surviving lethal ZIKV infection. Since virus mutations were not associated with outcome, a sex-specific host response likely explains the observed sex difference.

## 1. Introduction

Zika virus (ZIKV) is a pathogenic member of the *Flaviviridae* family and is transmitted to humans from *Aedes* mosquito vectors but also through bloodborne and mucosal routes, including sexual contact [1,2,3]. Since 2015, ZIKV has caused a series of outbreaks, with more than 1,000,000 estimated cases occurring in the Americas [4]. ZIKV is notable for its vertical transmission from mother-to-fetus and neurovirulence in fetal brain tissue, which has been associated with negative outcomes such as miscarriage and microcephaly [5,6]. ZIKV may also cause Guillain–Barré Syndrome, a severe autoimmune disorder affecting the peripheral nervous system [7,8,9]. There are currently no licensed therapies effective against ZIKV infection. Therefore, a safe and effective treatment for ZIKV disease is urgently required.

Favipiravir (also known as T-705) and ribavirin are antiviral compounds efficacious against a broad spectrum of RNA viruses [10,11]. Favipiravir and ribavirin are prodrugs, such that their primary mechanism of action occurs after they are phosphoribosylated into their active forms: favipiravir ribofuranosyl-5′-triphosphate (favipiravir-RTP) and ribavirin triphosphate (RTP), respectively [12,13,14]. This facilitates specific inhibition of the RNA polymerase or incorporation of the ribonucleoside analogs into the viral genome during replication, inducing virucidal mutagenesis [12,14,15,16]. Studies with other hemorrhagic fever-causing RNA viruses suggest that combined treatment with ribavirin and favipiravir may lead to increased efficacy, as low doses of ribavirin have been found to synergistically potentiate the antiviral activity of favipiravir in vivo [17].

Previous studies have demonstrated the anti-ZIKV activity of favipiravir and ribavirin in vitro [18,19,20,21]. Evaluation of the antiviral efficacy in vivo is typically performed in mice deficient of a proper type I interferon (IFN) response, as ZIKV does not readily cause severe disease in wild-type mice [22,23]. It was recently reported that ribavirin treatment delayed the onset of lethal ZIKV disease in male STAT1^−/−^ mice [24]. The purpose of this study was to evaluate the protective efficacy of favipiravir and ribavirin in interferon receptor α/β knockout (IFNAR^−/−^) mice, an established model for lethal ZIKV infection [25]. We found that favipiravir, but not ribavirin treatment, resulted in animals surviving the lethal infection. Unexpectedly, treatment worked much better in female mice (87% survival) compared to male mice (25% survival).

## 2. Materials and Methods

### 2.1. Ethics Statement

All infectious in vitro and in vivo work was performed in a biosafety level 2 laboratory at the Rocky Mountain Laboratories (RML), Division of Intramural Research (DIR), National Institute of Allergy and Infectious Disease (NIAID), National Institutes of Health (NIH) in Hamilton, MT. All animal work was approved by the Institutional Animal Care and Use Committee (IACUC) and performed according to the guidelines of the Association for Assessment and Accreditation of Laboratory Animal Care, International and our Office of Laboratory Animal Welfare (assurance number A4149-01). All procedures were carried out by certified personnel. Humane endpoint criteria in compliance with IACUC-approved scoring parameters were used to determine when animals should be humanely euthanized.

### 2.2. Cells and Viruses

C6/36 (*Aedes albopictus*) cells were grown at 32 °C and 5% CO_2_ in modified Eagle’s medium (MEM) (Thermo Fisher Scientific, Waltham, MA, USA) containing 10% FBS (Wisent), non-essential amino acids solution (Thermo Fisher Scientific, Waltham, MA, USA), 50 U/mL penicillin (Thermo Fisher Scientific, Waltham, MA, USA), and 50 μg/mL streptomycin (Thermo Fisher Scientific, Waltham, MA, USA) [25]. VeroE6 cells were grown at 37 °C and 5% CO_2_ in 10% FBS Dulbecco’s modified Eagle’s medium (DMEM) containing 2 mM L-glutamine (Thermo Fisher Scientific, Waltham, MA, USA), 50 U/mL penicillin (Thermo Fisher Scientific, Waltham, MA, USA), and 50 μg/mL streptomycin (Thermo Fisher Scientific, Waltham, MA, USA). Four strains of ZIKV were used in the studies: ZIKV-French Polynesia (human, 2013 [26]), ZIKV-Paraiba (human, Brazil 2015; GenBank accession number KX280026.1 [27]), ZIKV-PRVABC59 (human, Puerto Rico 2015; GenBank accession number KU501215), and ZIKV-MR766 (rhesus macaque, Uganda 1947 [28]). Viruses were propagated in C6/36 cells, titered on VeroE6 cells, and stored at −80 °C until use.

### 2.3. Drug Treatment

As the vehicle, we used 74.6 mg/mL United States Pharmacopeia (USP)-grade meglumine (Sigma, St. Louis, MO, USA) in phosphate-buffered saline. USP-grade ribavirin (Spectrum Chemical, Gardena, CA, USA) was dissolved in the vehicle to a final concentration of 15 mg/mL or 30 mg/mL for treatment with 150 mg/kg or 300 mg/kg, respectively. Favipiravir (Advanced Chem Blocks, Burlingame, CA, USA) was dissolved in the vehicle to a final concentration of 15 mg/mL or 30 mg/mL for treatment with 150 mg/kg or 300 mg/kg, respectively. Control groups in the study received the vehicle alone. Drugs were injected intraperitoneally (IP) in 0.2 mL per treatment.

### 2.4. In Vitro Antiviral Treatment

The antiviral efficacies of ribavirin and favipiravir were evaluated on VeroE6 cells infected with four different ZIKV isolates. ZIKV-MR766 was selected as a representative of the African lineage, ZIKV-French Polynesia as a representative of the Asian lineage prior to introduction to the Americas, ZIKV-Paraiba as a representative of the South American lineage associated with microcephaly, and ZIKV-PRVABC59 as a Puerto Rican lineage most relevant for potential ZIKV emergence in North America. To assess antiviral efficacy, VeroE6 cells were infected with the four ZIKV strains at an MOI of 0.1 in triplicate [25]. Inoculum was left on the cells for 1 h at 37 °C. After inoculation, the medium was replaced with 2% FBS DMEM containing 1 mM L-glutamine (Thermo Fisher Scientific, Waltham, MA, USA), 50 U/mL penicillin (Thermo Fisher Scientific, Waltham, MA, USA), 50 μg/mL streptomycin (Thermo Fisher Scientific, Waltham, MA, USA), and various concentrations of favipiravir and ribavirin (0, 1, 2, 5, 10, 25, 50, 100 μg/mL) diluted in 5 mg/mL meglumine. Cells were incubated for 72 h at 37 °C and 5% CO_2_. Samples of culture supernatants were subsequently taken and stored at −80 °C for quantification via median tissue culture infectious dose (TCID_50_) assay.

### 2.5. In Vivo IFNAR^−/−^ Mouse Studies

Groups of female and male C57BL/6 IFNAR^−/−^ mice, 6–9 weeks of age, received an IP injection of 1000 LD_50_ (5000 plaque-forming units (PFU)) or 100 LD_50_ (500 PFU) ZIKV-French Polynesia on day 0. Mice were treated with either vehicle-control, favipiravir 300 mg/kg, favipiravir 150 mg/kg, ribavirin 150 mg/kg, or a combination of favipiravir and ribavirin 150 mg/kg, as outlined in the respective experiment. Treatment started either at 8 h, 24 h, or 48 h post-infection (p.i.) with ZIKV, depending on the experiment, and was continued every 24 h for 14 days. Mice were monitored for weight loss and survival. In the final experiment, four mice per group were euthanized on day 3 and 7 p.i. for virus titration, sequencing, and pathology. Daily body weight was recorded until day 21 p.i., a time when the animals had fully recovered from the disease. All surviving mice were monitored until day 42 p.i.

### 2.6. Pathology, Immunohistochemistry, and In Situ Hybridization

Tissues were fixed in 10% neutral-buffered formalin for a minimum of 7 days. Fixed tissues were placed in cassettes and processed with a Sakura VIP-5 Tissue Tek on a 12 h automated schedule using a graded series of ethanol, xylene, and ParaPlast Extra. Embedded tissues were sectioned at 5 μm and dried overnight at 42 °C prior to staining with hematoxylin and eosin (H&E) or a rabbit polyclonal antibody for immunohistochemistry (IHC) against ZIKV NS2B (cat# GTX133308, Genetex, Irvine, CA, USA). Tissue samples were evaluated in detail with the following scoring system: 0 = no lesions; 1 = small number of necrotic cells; 2 = moderate necrosis; 3 = significant necrosis; 4 = coalescing necrosis; 5 = diffuse necrosis. In situ hybridization (ISH) was performed with the V-ZIKA-pp-02 probe for ZIKV (ACDBIO, Newark, CA, USA), as previously described [25].

### 2.7. ZIKV Sample Titrations by TCID_50_ Assay

Cell culture supernatant and blood samples were thawed, and 10-fold serial dilutions were prepared. Tissue samples were homogenized with 1 mL of DMEM without additives and a stainless-steel bead at 30 Hz for 10 min using a TissueLyser II (Qiagen, Germantown, MD, USA). Tissue debris was sedimented at 8000 rpm for 10 min and 10-fold serial dilutions were prepared. All dilutions were inoculated onto a confluent layer of VeroE6 cells in a 96-well plate in triplicates. After 72 h, the cytopathic effect caused by ZIKV in cells was recorded and the TCID_50_ was calculated for each sample, employing the Reed and Muench method [29].

### 2.8. Quantitative RT-PCR

RNA extraction from whole blood samples was performed with the QiAmp Viral RNA Kit (Qiagen); tissue samples were homogenized with stainless steel beads before proceeding with the RNeasy Mini Kit (Qiagen, Germantown, MD, USA). Real-time quantitative PCR reactions were performed with the QuantiFast ProbeRT-PCR enzyme and master mix (Qiagen, Germantown, MD, USA) on a RotorGene Q thermal cycler (Qiagen, Germantown, MD, USA) following the manufacturer’s instructions, as previously described [25]. ZIKV-French Polynesia-specific primers and probe targeting NS5 were used: forward (5′-TTGGTCATGATACTGCTGATTGC), reverse (5′-CCTTCCACAAAGTCCCTATTGC), probe (5′-CGGCATATAGCATCAGGTGCATAGGAG). Absolute quantification of genome equivalents per mg or mL was performed using in vitro transcribed ZIKV RNA copy number standards, determined by droplet digital PCR and a three-point 10-fold serial dilution, run in parallel.

### 2.9. Next-Generation Sequencing

Brain, spleen, and gonad tissue samples were homogenized, and total RNA extracted with RNeasy Mini Kit (Qiagen, Germantown, MD, USA). Total RNA was extracted from whole blood samples and challenge virus stock using the QIAamp Viral RNA Mini Kit (Qiagen, Germantown, MD, USA). RNA was then prepared with rRNA depletion. First- and second-strand cDNA synthesis was performed using SuperScript III First-Strand Synthesis SuperMix (Thermo Fisher Scientific, Waltham, MA, USA) and Phusion PCR Master Mix with GC Buffer (NEB, Ipswich, MA, USA) with purification between each strand synthesis using QIAquick PCR Purification Kit (Qiagen, Germantown, MD, USA). cDNA was indexed using the Nextera XT DNA library preparation kit and index kit v2 (Illumina, San Diego, CA, USA). Normalization, library pooling, and denaturation were performed according to Illumina protocols and run on the MiSeq (Illumina, San Diego, CA, USA). Sequences were aligned to the stock strain of ZIKV-French Polynesia (human, 2013 [26]). SNP analysis was performed with minimum coverage = 10 and minimum variant frequency = 0.1.

### 2.10. EC_50_ Calculation and Statistical Analysis

The half-maximal effective concentrations (EC_50_) of ribavirin and favipiravir against in vitro ZIKV propagation on VeroE6 cells were calculated using nonlinear regression and the least squares fit method (Table S1). Confidence intervals (CIs) of 95% were computed using the likelihood ratio asymmetric method. As 95% CIs could not be determined for all the examined strains due to high variance and limited sample size, only the 95% CI for the combined ZIKV dataset was reported. Survival curves for all animal experiments were analyzed for statistical significance with the Mantel–Cox test. All ZIKV titer and RT-qPCR results were analyzed with two-way ANOVA. All significantly different results are indicated in the respective figures (* *p* ≤ 0.05, ** *p* ≤ 0.01, *** *p* ≤ 0.001, **** *p* ≤ 0.0001).

## 3. Results

### 3.1. Efficacy of Ribavirin and Favipiravir Treatment of ZIKV Infection In Vitro

**Figure 1:**
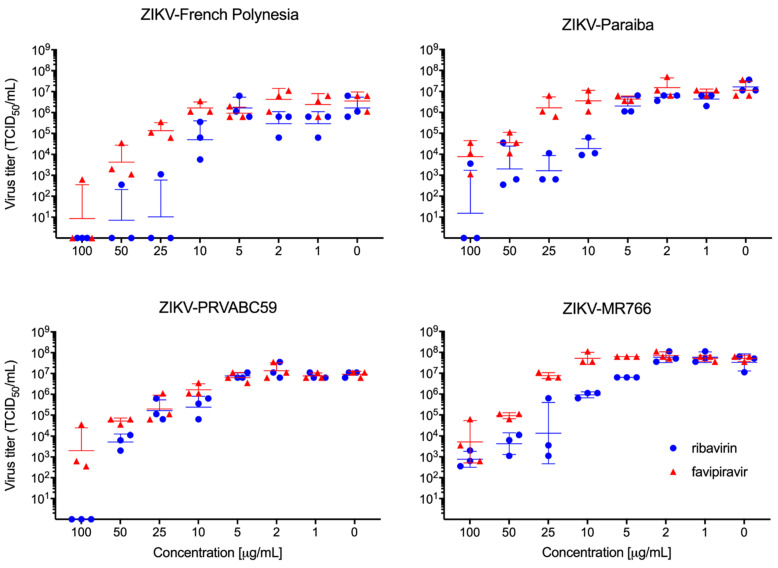
In vitro ribavirin and favipiravir efficacy. Vero E6 cells were infected in triplicate with four different strains of ZIKV and treated with different concentrations of favipiravir and ribavirin. Virus titration was performed to determine antiviral efficacy against ZIKV-French Polynesia, ZIKV-Paraiba, ZIKV-PRVABC59, and ZIKV-MR766 using median tissue culture infectious dose (TCID_50_) assay. Geometric mean and standard deviation are shown.

VeroE6 cell infections resulted in a similar dose-dependent response for ribavirin and favipiravir treatment for each of the four ZIKV strains tested (Figure 1). Among the individual strains, EC_50_ values ranged from 0.4825 to 9.431 µg/mL for ribavirin and from 6.934 to 34.73 µg/mL for favipiravir (Table S1). While we cannot exclude the possibility that cells treated with 100 μg/mL ribavirin may have exhibited some degree of cytotoxicity (Figure 1), favipiravir did not result in apparent cytotoxicity at any of the tested concentrations.

### 3.2. Ribavirin and Favipiravir Treatment of ZIKV Infection in IFNAR^−/−^ Mice

**Figure 2:**
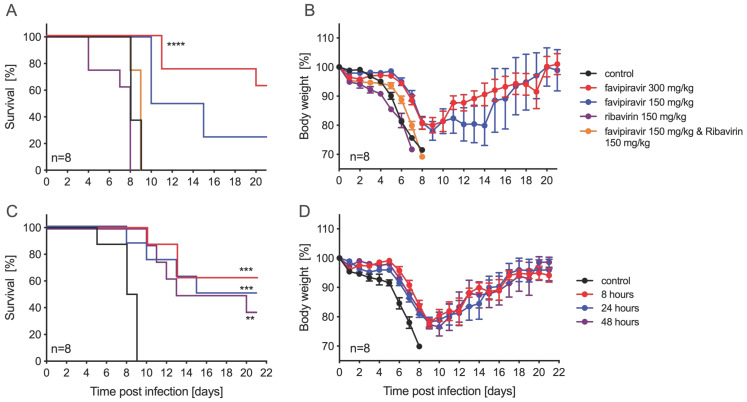
Ribavirin and favipiravir treatment efficacy against ZIKV-French Polynesia in IFNAR^−/−^ mice. Female and male mice (6–9 weeks old) were infected with 1000 median lethal doses (LD_50_) (5000 plaque-forming units (PFU)) ZIKV-French Polynesia and treated with antivirals starting 8 h post-infection and continuing treatment every 24 h until day 14. (**A**) Survival and (**B**) body weight changes for each treatment group (n = 8) and vehicle-control group (n = 8) are shown. Female and male mice were infected with 100 LD_50_ (500 PFU) and treated with 300 mg/kg favipiravir starting 8 h, 24 h, or 48 h post-infection, and continued daily treatment until day 14. (**C**) Survival and (**D**) body weight changes in treated (n = 8) and vehicle-control groups (n = 8) are shown. Error bars indicate standard deviation. Statistically significant results are indicated as follows: ** *p* < 0.01, *** *p* < 0.001, **** *p* < 0.0001.

Next, we evaluated the efficacy of ribavirin and favipiravir antiviral treatment in male and female IFNAR^−/−^ mice. For this, we decided to use ZIKV-French Polynesia as both compounds, showing relatively strong and comparable in vitro effects against this isolate (Figure 1 and Table S1), and this isolate caused the most consistent pathogenic phenotype in the IFNAR^−/−^ mouse model, as previously shown [25]. Other parameters regarding the infection, including the selection of IP as the route of infection, were based upon a previously established lethal mouse model of ZIKV infection [25]. Previous studies using both inhibitors against Crimean–Congo hemorrhagic fever virus (CCHFV) in the lab established treatment concentrations and time points [30]. Groups of eight mice received 1000 LD_50_ ZIKV-French Polynesia IP at day 0. Treatment with vehicle alone (control), ribavirin, favipiravir, or a combination of both antivirals was performed at different doses IP starting 8 h p.i. and continuing every 24 h until day 14 p.i. The control group, along with mice treated with ribavirin (150 mg/kg) or the combination of ribavirin and favipiravir (150 mg/kg each), all lost weight and succumbed to disease by days 8–9 p.i. (Figure 2A,B). Mice treated with favipiravir at 300 mg/kg or 150 mg/kg became sick, as evidenced by weight loss, but recovered, resulting in survival rates of 62.5% or 25%, respectively (Figure 2A). Favipiravir at 300 mg/kg was next evaluated for delayed treatment start using a lower ZIKV infection dose. Groups of eight IFNAR^−/−^ mice were IP-infected with 100 LD_50_ ZIKV-French Polynesia and then treated with favipiravir at 300 mg/kg or vehicle starting at 8 h, 24 h, or 48 h p.i. Treatment continued every 24 h until day 14 p.i. All animals in the treatment and control groups lost weight, but only the control animals uniformly succumbed to disease between 5–9 days p.i. (Figure 2C,D). The group that started treatment at 8 h p.i. had the highest survival rate of 62.5% (Figure 2C). In the 24 h and 48 h treatment start groups, there were 50% and 37.5% animals surviving, respectively (Figure 2C). Independent of the challenge dose, treatment start at 8 h resulted in the highest survival rates. 

### 3.3. Sex-Specific Difference with Favipiravir Treatment

Further analysis of the survival by sex in the delayed treatment study revealed a sex-specific difference in response to favipiravir treatment. When treated with 300 mg/kg favipiravir starting at 8 h p.i., 100% (5/5) of female mice survived, compared to 0% (0/3) of male mice (Figure S1A). The animals in the control group, consisting of four female and four male mice, all succumbed to disease by day 9 (Figure S1A). Looking back, this phenomenon was also observed in the first mouse experiment for the 300 mg/kg favipiravir group. All animals in the control group, consisting of five females and three males, succumbed to disease by day 9 (Figure S1B). For the 300 mg/kg favipiravir treatment group, 100% (4/4) of females survived, compared to 25% (1/4) of males that survived (Figure S1B).

**Figure 3:**
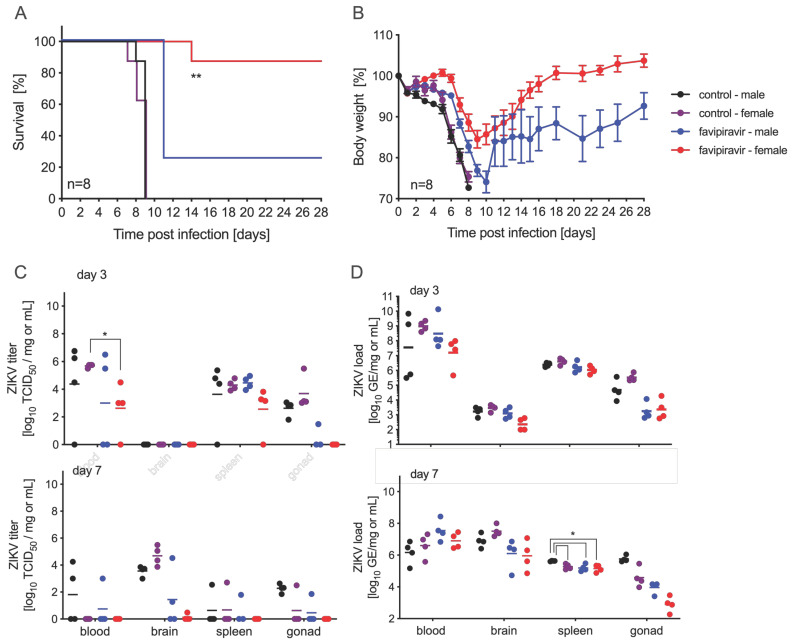
Sex differences in favipiravir-treated and ZIKV-infected IFNAR^−/−^ mice. Female and male mice (6–9 weeks old) were infected with 100 LD_50_ (500 PFU) ZIKV-French Polynesia and treated with 300 mg/kg favipiravir starting 8 h post-infection and continuing treatment every 24 h until day 14. (**A**) Survival and (**B**) body weight changes for each treatment group (n = 8) and vehicle-control group (n = 8) are shown. Error bars indicate standard deviation. On days 3 and 7 after infection, blood and tissue samples were taken to determine (**C**) ZIKV titer (n = 4) by using median tissue culture infectious dose (TCID_50_) assay and (**D**) ZIKV load (n = 4). Geometric mean is depicted. Statistical significance is indicated as follows: * *p* < 0.05, ** *p* < 0.01.

In order to further investigate this sex bias with favipiravir treatment in this mouse model, we performed another experiment with four groups of 16 IFNAR^−/−^ mice, with one control and one treatment group for each sex. Mice were infected with 100 LD_50_ ZIKV-French Polynesia on day 0 and treated with 300 mg/kg favipiravir 8 h p.i. and every 24 h until day 14 p.i. Eight mice in each group were observed for body weight loss and survival and monitored for 42 days p.i. As in the previous experiments, all control male and female mice succumbed to disease by day 9 (Figure 3A). While all treated male and female animals lost weight, 25% (2/8) of males survived compared to 87.5% (7/8) of females (Figure 3A,B). Four mice in each group were euthanized on days 3 and 7 p.i. for virus load determination and pathology. While ZIKV RNA was present in all blood, brain, spleen, and gonad samples tested, virus was isolated from many, but not all, of the samples (Figure 3C,D). We did not observe a sex-based difference in ZIKV RNA or titer except for treated female samples collected on day 7 p.i., where we only could isolate ZIKV from a single tissue sample, compared to more in the control female and treated male groups (Figure 3C).

**Figure 4:**
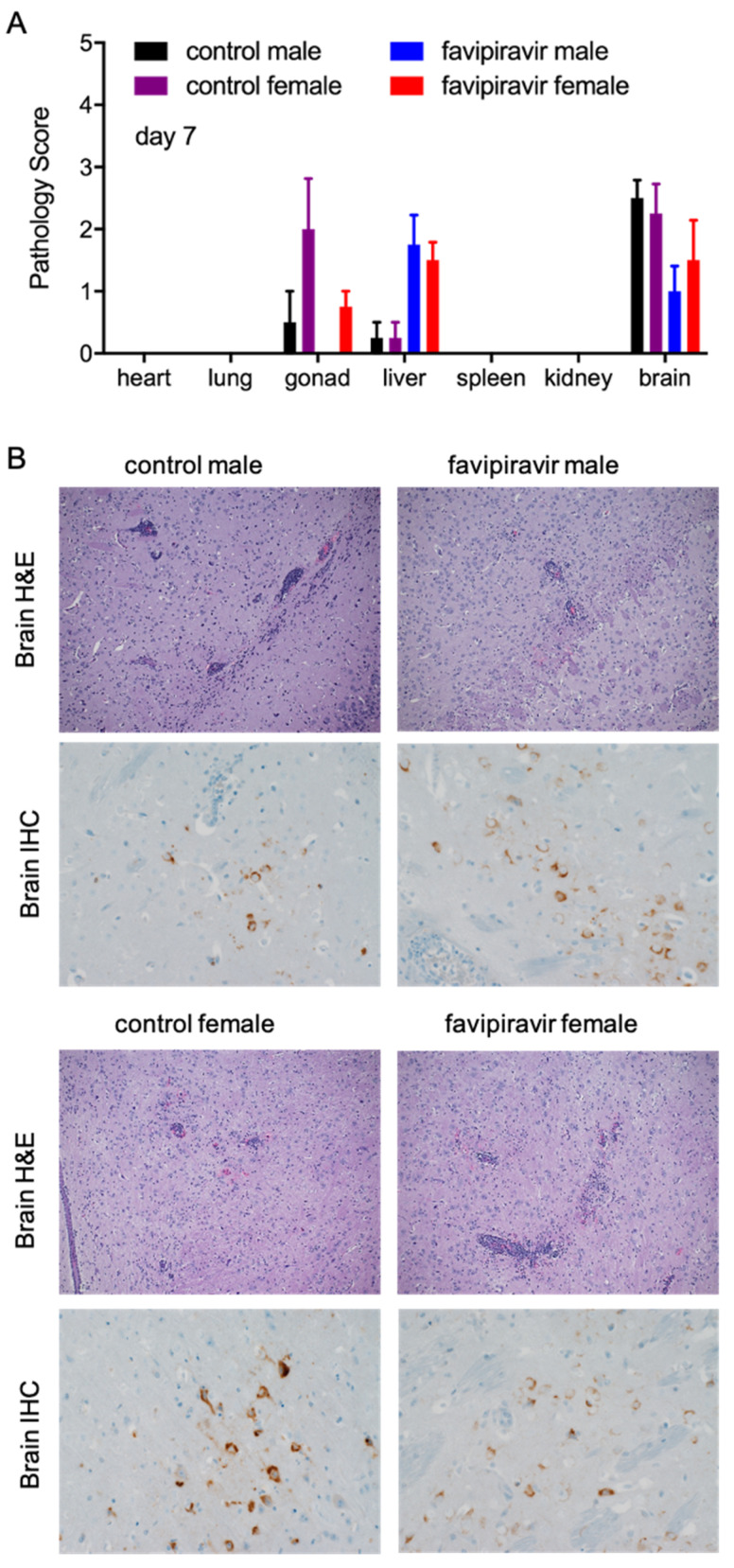
Pathological analysis of tissue samples from favipiravir-treated, ZIKV-infected IFNAR^−/−^ mice. Female and male mice (6–9 weeks old) were infected with 100 LD_50_ (500 PFU) ZIKV-French Polynesia and treated with 300 mg/kg favipiravir starting 8 h post-infection and continuing treatment every 24 h until day 14. Tissue samples were collected from four mice per group on day 7 post-infection. (**A**) Tissues were scored for pathology using the following scoring system: 0 = no lesions; 1 = small number of necrotic cells; 2 = moderate necrosis; 3 = significant necrosis; 4 = coalescing necrosis; 5 = diffuse necrosis. Error bars indicate standard deviation. (**B**) Brain tissue fixed with formalin was stained with hematoxylin and eosin (H&E) and imaged at 100× or stained for ZIKV NS2B antigen using immunohistochemistry (IHC) and imaged at 400× magnification. Arrows indicate meningoencephalitis (perivascular cuffing); arrowheads indicate gliosis.

Pathological changes in tissue samples collected on day 7 p.i. from treated and control mice were scored as outlined in the methods (Figure 4A). No significant differences were noted between male and female control and treated mice. The increased liver pathology score for treated mice was due to the treatment with favipiravir and has previously been observed [27]. However, there was a slight increase in the severity of histopathological changes with the untreated groups when analyzed by immunohistochemistry (IHC) and in situ hybridization (ISH). Animals in all groups developed some degree of meningoencephalitis that is characterized by multifocal expansion of the meninges and Virchow–Robin’s space by small to moderate numbers of lymphocytes and fewer macrophages and neutrophils (Figure 4B). There was necrotic cellular debris admixed with these inflammatory cells. Additionally, there was gliosis in the adjacent parenchyma, along with small amounts of necrotic cellular debris and rare neutrophils and multifocal necrotic neurons (Figure 4B). By day 7 p.i., all 16 control mice developed encephalitis that was generally mild to moderate in severity (Figure 4B). The treated mice, both males and females, also developed encephalitis, but only 1/8 animals displayed lesions of moderate severity; 5/8 mice had minimal to mild lesions and 2/8 mice were essentially normal. In addition to the brain lesions, changes were noted in the reproductive organs of both sexes (Figure S2). Four male mice presented with pathology characteristic of epididymitis and one of those mice in the untreated control group demonstrated multifocal necrosis of epithelial lining cells (Figure S2A). Likewise, 5/8 female mice, treated and control, had necrosis of granulosa cells within primary follicles (Figure S2B).

The possibility of favipiravir acting in a sex-specific manner has not been reported previously, but one potential mechanism could be a sex-specific host pressure to select for an accumulation of mutations in the viral genome. ZIKV isolated from blood, brain, spleen, and gonad samples was analyzed with next-generation sequencing and compared to the sequence of ZIKV-French Polynesia initially used to inoculate the IFNAR^−/−^ mice. Notably, not all tissue samples collected from each mouse in each group had enough virus present to determine the sequence. Single-nucleotide polymorphisms (SNPs) were detected in day 7 brain tissue samples from treated and control male and female mice (Table S2). We were able to obtain sequences from all eight control animals, but only from 3/4 treated males and 2/4 treated females. SNPs were only detected in spleen and gonad tissue samples (day 3 and day 7) in all control mice (Tables S3 and S4) as we were not able to amplify ZIKV sequences from the treated animal samples. There were also SNPs identified in blood samples (day 3 and day 7) from all groups (Table S5), but to a much lesser extent than the brain. Overall, there were no significant mutations that correlated with the difference in survival between males and females, nor was there even a correlation between control and favipiravir-treated animals.

## 4. Discussion

In this study, we assessed the efficacy of ribavirin and favipiravir antiviral treatments against ZIKV infection. Our cell culture experiments extended previously published results by others where ribavirin and favipiravir inhibited ZIKV-MR766 and ZIKV-PRVABC59 replication [18]. We expanded the panel of tested ZIKV isolates and showed that both antivirals are similarly effective against ZIKV-French Polynesia and ZIKV-Paraiba (Figure 1 and Table S1). Similar to previous findings, we observed an anti-ZIKV effect for both compounds [18,19,20,21]. In our dataset, ribavirin exhibited a lower EC_50_ than favipiravir under the in vitro conditions examined (Table S1). However, when tested in IFNAR^−/−^ mice, ribavirin did not protect mice from lethal ZIKV challenge, as shown before with male STAT1^−/−^ mice [24]. In contrast, daily administration of 300 mg/kg favipiravir protected the IFNAR^−/−^ mice from lethal ZIKV challenge (Figure 2). This is consistent with results from another flavivirus, yellow fever virus, where favipiravir improved disease outcome in the hamster model [30]. Additionally, favipiravir, but not ribavirin, is an effective treatment against other RNA virus infections, such as CCHFV, for which it has been shown to protect mice and macaques from lethal disease [31,32]. This may be due to higher mutagenic activity of favipiravir compared to ribavirin [33]. Interestingly, favipiravir alone or in combination with ribavirin has been described to protect mice from lethal Lassa virus (LASV) infection [34], and favipiravir has also successfully been used as a treatment for LASV in macaques [35]. We speculate that distinct mechanisms of action reported for ribavirin and favipiravir on intracellular nucleotide pools [36] may mitigate or worsen disease in a virus-specific manner. Furthermore, a combination treatment with low doses of ribavirin and favipiravir showed reduced protection in our model compared to ribavirin alone (Figure 2). Based on this finding, we would hypothesize that ribavirin-induced depletion of GTP levels may exacerbate ZIKV disease pathology.

Importantly, in our experiments we used male and female mice. We have shown here for the first time that favipiravir can indeed be a viable treatment option against lethal ZIKV infection in female IFNAR^−/−^ mice. Others have only used male STAT1^−/−^ mice and observed no survival in these animals [24]. We would have missed this result if only male mice were used for the experiment. Among the mice that exhibited signs of disease, there was no significant difference in pathology between the male and female treatment groups. To investigate a potential mechanism of this sex bias, we sequenced the ZIKV isolated from brain, blood, spleen, and gonad tissue of treated and untreated animals and compared them to the challenge virus stock sequence. There were no specific mutations from tissues of infected mice that correlated with the sex-specific survival discrepancy, confirming that the outcome was not due to the presence of attenuating virus variants. These results leave us to speculate that sex-specific host responses might play an important role. Indeed, it has previously been shown that the type I interferon response promotes reduced viral disease pathology in male mice, compared to females, suggesting that the use of a mouse model deficient in type I interferon responses may be a relevant factor [37]. Interestingly, neurological problems as a consequence of ZIKV infection in utero have been associated with different testosterone levels in male offspring [38]; however, we were not able to analyze hormone levels in our mice.

Until now, there have been no studies reporting a sex bias response to favipiravir treatment for viral infection. A study, using mixed-sex IFNAR^−/−^ mice, of favipiravir and ribavirin against CCHFV infection did not observe a sex-specific difference in treatment efficacy [30]. Favipiravir is highly potent against Rift Valley fever virus and CCHFV, but in previous studies only female hamsters and female IFNAR^−/−^ mice, respectively, were used [39,40]. Additionally, an efficacy study for ribavirin and favipiravir combination treatment only tested one sex for guinea pig (male) and hamster (female) experiments challenging against Junin and Pichinde hemorrhagic fever viruses, respectively [17]. There have been previous reports indicating potential sex differences in humans in response to antiviral treatment with other nucleoside analogs [41,42]. A recent study by Hanioka et al. suggests that favipiravir exhibits markedly variable species- and sex-dependent rates of biotransformation into inactive M1. Congruent with our data, Hanioka et al. report that favipiravir drug clearance was more than four-fold higher in liver cytosolic fractions from male mice than those from female mice [43]. Such large differences in pharmacokinetics would likely justify sex-based adjustments in the dosing regimen for future experiments. While our findings elucidate a sex-biased response to treatment with favipiravir, further investigation is necessary to explicitly determine to what degree this is a result of sex-dependent differences in ZIKV disease, the IFNAR^−/−^ model, or favipiravir activity.

In conclusion, we have shown that favipiravir is effective at inhibiting ZIKV replication and increases survival in the IFNAR^−/−^ mouse model. Most importantly, favipiravir protected female mice (87.5%) at a much higher rate than male mice (25%). This highlights the importance of using both male and female animals for in vivo efficacy studies. While further efficacy and safety testing is necessary, favipiravir may be considered as a potential therapeutic for human ZIKV cases or those with high-risk exposure to ZIKV. As the replication cycle of flaviviruses is similar, favipiravir may also be a potential therapeutic option for diseases caused by other emerging and re-emerging human–pathogenic flaviviruses such as Dengue virus, West Nile virus, and Kyasanur Forest disease. Further studies are needed to confirm this hypothesis.

## Data Availability

Data presented in this study are available in the supplemental materials and upon request from the corresponding author.

## Supplementary Materials

The following are available online at https://www.mdpi.com/article/10.3390/microorganisms9061178/s1. Figure S1. Gender differences in favipiravir-treated and ZIKV-infected IFNAR^−/−^ mice. Figure S2. Pathological analysis of tissue samples from favipiravir-treated and ZIKV-infected IFNAR^−/−^ mice. Table S1. EC_50_ of inhibitors against ZIKV. Table S2. SNPs found in the brain of control and favipiravir-treated mice. Table S3. SNPs found in the spleen of control and favipiravir-treated mice. Table S4. SNPs found in the gonads of control and favipiravir-treated mice. Table S5. SNPs found in the blood of control and favipiravir-treated mice.
