# Supplementary material for: Favipiravir (T-705) Protects IFNAR−/− Mice against Lethal Zika Virus Infection in a Sex-Dependent Manner
Source: Microorganisms. 2021 May 29;9(6):1178. doi: 10.3390/microorganisms9061178 (PMC8227069; doi:10.3390/microorganisms9061178)
Supplement: Supplementary file 1 [file microorganisms-09-01178-s001.zip › microorganisms-1221711-supplementary.pdf]

Favipiravir (T-705) protects IFNAR$^{-/-}$ mice against lethal Zika virus infection in a sex-dependent manner

Keesha Matz[1§], Jackson Emanuel[1§#], Julie Callison[1], Don Gardner[2], Rebecca Rosenke[2], Reinaldo Mercado-Hernandez[1], Brandi N. Williamson[1], Heinz Feldmann[1], and Andrea Marzi[1*]

[1]Laboratory of Virology, and [2]Rocky Mountain Veterinary Branch, Division of Intramural Research, National Institute of Allergy and Infectious Diseases, National Institutes of Health, Hamilton, MT, USA

[§]Authors contributed equally.

Supplementary Materials
Figures S1, S2
Tables S1-S5

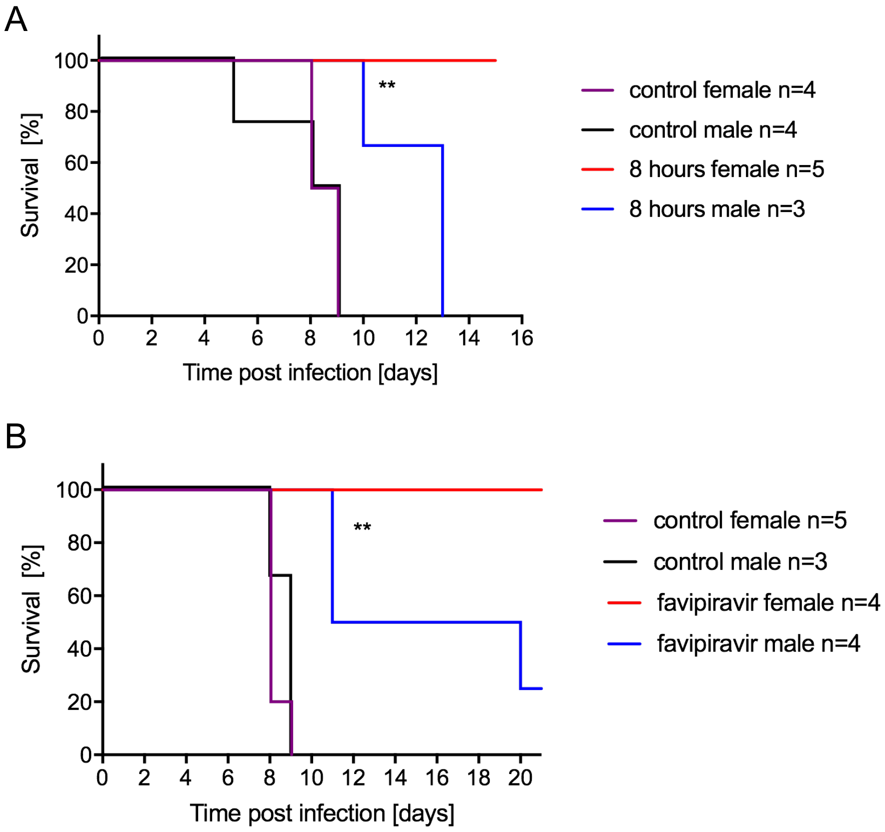

Figure S1. Gender differences in favipiravir-treated and ZIKV-infected IFNAR$^{-/-}$ mice. IFNAR$^{-/-}$ mice (6-9 weeks old) were infected with ZIKV-French Polynesia treated with 300 mg/kg favipiravir starting 8 hr post-infection and every 24 hrs until day 14. (A) Survival curve for males and females from 8 hr and control groups from Fig. 2C. (B) Survival curve for males and females from favipiravir 300 mg/kg and control groups from Fig. 2A. Statistical significance is indicated as follows: ** $p < 0.01$.

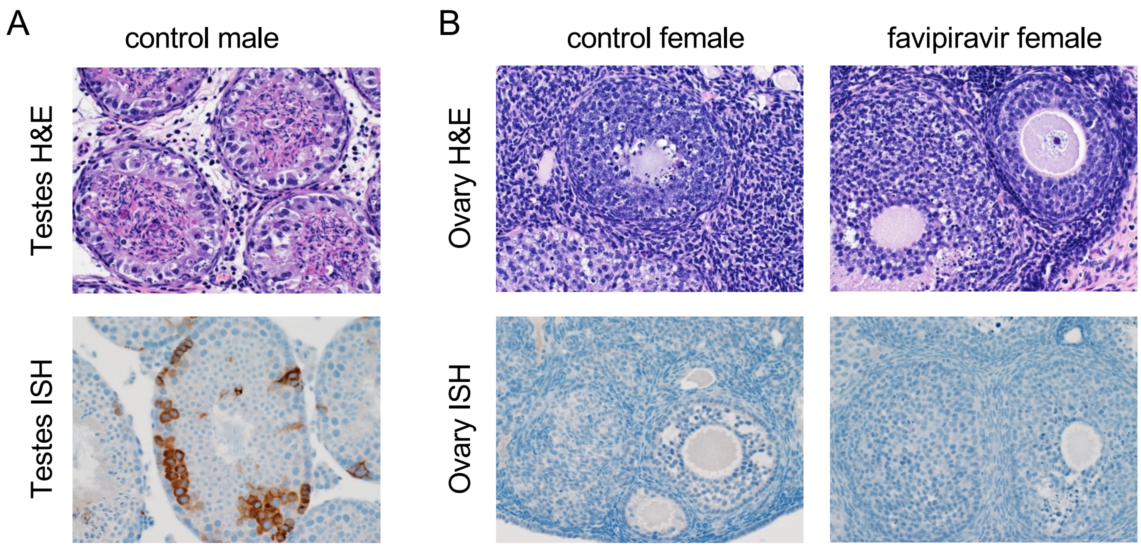

Figure S2. Pathological analysis of tissue samples from favipiravir-treated and ZIKV-infected IFNAR$^{-/-}$ mice. IFNAR$^{-/-}$ mice were infected with 100 LD_{50} (500 PFU) ZIKV-French Polynesia and treated with 300 mg/kg favipiravir 8 hrs post-infection and every 24 hrs until day 14. Tissue samples were collected on day 7 post-infection, fixed with formalin, stained with hematoxylin and eosin (H&E) or probed for ZIKV using *in situ* hybridization (ISH), and imaged at 400x magnification. (A) Vehicle-control treated male mouse testes and epididymis; (B) Vehicle-control and favipiravir-treated female mouse ovary.

Table S1. EC_{50} of inhibitors against ZIKV.

**Virus**	**Ribavirin EC_{50} (95% CI) [µg / ml]**	**Favipiravir EC_{50} (95% CI) [µg / ml]**
**ZIKV**	4.749 (1.355-22.33)	23.34 (4.246-875.5)
**ZIKV-MR766**	5.209	34.73
**ZIKV-PRVABC59**	9.431	10.22
**ZIKV-Paraiba**	0.4825	7.152
**ZIKV-French Polynesia**	6.047	6.934

Table S2. SNPs found in the brain of control and favipiravir-treated mice.

Brain							
		Samples successfully sequenced		4/4 animals	3/4 animals	4/4 animals	2/4 animals
				male		female	
				control	favipiravir	control	favipiravir
				day 7	day 7	day 7	day 7
gene	nt location	nt change	amino acid change				
C	376	G -> A	A90T	11.90%			
pr	538	G -> A	A144T			14.60%	
pr	550	C -> T	P148S			11.90%	
pr	722	A -> G	H205R	11.10%			
M	872	C -> T	P255L				10.70%
M	927	C -> T	synonymous			16.9%, 14.20%	
E	1,051	T -> C	synonymous	10.80%			
E	1,093	C -> T	P329S		10.80%		
E	1,242	T -> C	synonymous	38.10%			
E	1,292	G -> A	C395Y			11.10%	
E	1,427	G -> A	G440E	23.80%, 10.30%		69.00%, 45.40%, 30.80%	
E	1,808	T -> C	M567T		15.80%		
E	2,078	A -> T	E657V	22.20%			
E	2,157	G -> A	synonymous	35.70%			
NS1	2,619	A -> C	synonymous			20.80%	
NS1	2,725	C -> A	L873M	16.70%			
NS1	2,786	G -> A	R893K	16.20%			
NS1	2,931	T -> A	H941Q			30.80%	
NS1	2,997	C -> T	synonymous	58.30%	83.30%	37.1%, 36.50%	60.00%
NS1	3,243	C -> T	synonymous				28.60%
NS1	3,459	C -> T	synonymous	39.4%, 19.10%			
NS2A	3,875	C -> T	A1256V				12.20%
NS2A	3,896	C -> T	A1263V	40%, 63.60%, 23.40%	100%, 41.70%	44.60%, 87.30%, 45.90%, 70%	83.30%
NS2A	3,993	C -> T	synonymous		84.60%		
NS2B	4,293	G -> A	synonymous		18.80%		
NS2B	4,323	C -> T	synonymous			20.70%	
NS2B	4,357	T -> G	S1417A	32.10%, 21.30%, 36.80%	40.30%	42.70%, 36.40%, 10.00%	38.20%, 81%
NS3	4,650	A -> G	synonymous				47.10%
NS3	5,058	A -> T	synonymous				25.00%
NS3	5,190	A -> G	synonymous				50.20%
NS3	5,451	T -> C	synonymous			14.00%	
NS3	5,718	C -> T	synonymous			21.20%	
NS3	5,865	C -> T	synonymous	38.90%			
NS3	6,099	C -> T	synonymous	28.60%			
NS4A	6,585	T -> C	synonymous		17.10%		
NS4A	6,678	C -> T	synonymous	24.40%			
NS4A	6,680	T -> C	L2191S			12.90%	
2K	6,885	A -> G	synonymous				40.00%
NS4B	6,948	T -> A	S2280R	12.10%			
NS5	7,900	A -> G	I2598V				45.50%
NS5	8,106	T -> C	synonymous			10.30%	
NS5	8,313	T -> A	synonymous	48.40%			
NS5	8,472	C -> T	synonymous			16.90%	
NS5	8,490	C -> T	synonymous		45.50%		
NS5	8,535	C -> T	synonymous		19.00%		
NS5	8,694	A -> G	I2862M	11.60%			
NS5	9,167	G -> A	G3020E				12.50%
NS5	9,411	C -> T	synonymous			20.90%	
NS5	10,121	T -> A	V3338E		42.90%		
NS5	10,345	T -> C	synonymous				81.80%

Table S3. SNPs found in the spleen of control and favipiravir-treated mice.

Spleen							
		Samples successfully sequenced:		4/4 animals	0/4 animals	2/4 animals	0/4 animals
				male		female	
				control	favipiravir	control	favipiravir
				day 3	n/a	day 3	n/a
gene	nt location	nt change	amino acid change				
M	799	T -> C	S231P	15.50%, 14.90%			
E	1,427	G -> A	G440E	33.70%, 21.60%, 39.40%		14.50%, 24.70%	
NS1	2,593	C -> A	H829N	17.60%			
NS1	2,928	A -> C	K940N			10.90%	
NS1	2,997	C -> T	synonymous	10.10%		16.40%	
NS1	3,150	G -> A	M1014I			16.00%	
NS1	3,283	A -> G	K1059E	16.70%			
NS2A	3,896	C -> T	A1263V	23.90%, 12%, 36.20%		35.20%, 11.20%	
NS2B	4,357	T -> A	S1417T	20%, 10.60%			
NS2B	4,357	T -> G	S1417A	24.70%, 11.80%		16.30%	
NS3	6,373	A -> C	K2089Q	20.40%, 11.50%			
NS5	7,941	C -> T	synonymous	16.70%			
NS5	8,238	T -> C	synonymous	38.2%, 15.40%			
NS5	9,858	C -> T	synonymous	10.00%			

Table S4. SNPs found in the gonads of control and favipiravir-treated mice.

Gonad							
		Samples successfully sequenced:		1/4 animals	0/4 animals	4/4 animals	0/4 animals
				**male**		**female**	
				**control**	**favipiravir**	**control**	**favipiravir**
				day 3 & 7	n/a	day 3	n/a
gene	nt location	nt change	amino acid change				
C	391	A -> G	I95V	12.50%			
pr	722	A -> G	H205R			28.60%	
E	1,140	G -> A	synonymous			10.10%	
E	1,331	A -> G	K408R			10.20%	
E	1,344	C -> T	synonymous	14.80%			
E	1,427	G -> A	G440E			30.70%, 42.90%	
E	2,139	T -> C	synonymous			20.00%	
E	2,170	C -> T	H688Y			18.80%	
NS1	2,758	G -> A	V884M			11.40%	
NS1	2,768	T -> C	V887A			20.50%	
NS1	3,150	G -> A	M1014I			14.20%	
NS2A	3,888	G -> A	synonymous	24.00%			
NS2A	3,896	C -> T	A1263V			18.10%	
NS2A	3,900	C -> T	synonymous			13.00%	
NS2A	4,167	G -> A	synonymous	10.30%			
NS2B	4,357	T -> G	S1417A	64.30%		16%	
NS2B	4,439	C -> T	P1444L	11.40%			
NS3	5,748	G -> A	synonymous	11.10%			
NS3	5,865	C -> T	synonymous	38.90%			
NS3	5,958	C -> T	synonymous	18.20%			
NS3	6,007	A -> G	N1967D	10%			
NS3	6,277	A -> G	T2057A			12.80%	
NS3	6,365	A -> G	Y2086C			31.60%	
NS3	6,373	A -> C	K2089Q	19.40%		33.30%	
NS3	6,447	G -> A	synonymous			10.20%	
NS4A	6,768	G -> A	synonymous			12.90%	
NS4B	7,570	A -> G	T2488A	11.10%			
NS5	7,776	C -> A	truncation			28.60%	
NS5	7,829	C -> T	A2574V			40%	
NS5	7,882	C -> T	truncation			54.50%	
NS5	8,613	A -> G	synonymous			12.90%	
NS5	8,833	C -> T	H2909Y			36.40%	
NS5	9,618	G -> A	synonymous			20.00%	

Table S5. SNPs found in the blood of control and favipiravir-treated mice.

Blood							
		TSamples successfully sequenced:		2/4 animals	1/4 animals	4/4 animals	1/4 animals
				**male**		**female**	
				**control**	**favipiravir**	**control**	**favipiravir**
				day 3	day 3 & 7	day 3	day 3
gene	nt location	nt change	amino acid change				
C	251	T -> C	L48S		11.90%		
C	308	A -> G	N67S	25.00%			
E	1,427	G -> A	G440E	21.60%, 35.90%	25.90%	30.40%	
E	1,446	A -> G	synonymous	10.70%			
E	1,986	T -> C	synonymous	18.20%			
NS1	2,926	A -> G	K940E			16.70%	
NS1	2,997	C -> T	synonymous	25.00%	44%, 33.30%	24.0%, 15.20%	44.00%
NS2A	3,896	C -> T	A1263V	33.30%, 40.70%	59.40%	16%	41.70%
NS2A	4,101	G -> T	synonymous			30.80%	
NS2B	4,357	T -> A	S1417T	18.40%, 12.30%			
NS2B	4,357	T -> G	S1417A	20.40%, 20%	38.10%	33.30%, 20.00%	34.60%
NS3	5,570	C -> T	P1821L	10%			
NS3	6,365	A -> G	Y2086C			28.60%	
NS3	6,373	A -> C	K2089Q			42.90%	
NS5	7,518	T -> C	synonymous		11.80%		
NS5	8,238	T -> C	synonymous	36.40%			
NS5	8,344	A -> G	K2746E	13%			
NS5	9,041	A -> G	K2978R			12.10%	
NS5	9,114	T -> C	synonymous			11.40%	
